# Nurses as educators in the comprehensive heart failure care programme—Are we ready for it?

**DOI:** 10.1002/nop2.507

**Published:** 2020-05-19

**Authors:** Dorota Krówczyńska, Beata Jankowska‐Polańska

**Affiliations:** ^1^ The Cardinal Stefan Wyszyński Institute of Cardiology Warsaw Poland; ^2^ Department of Clinical Nursing Faculty of Health Sciences Wroclaw Medical University Wroclaw Poland

**Keywords:** education, heart failure, nurse, nursing

## Abstract

**Aim:**

To assess education frequency and nurses' comfort when educating patients hospitalized in different hospital units to prepare them for self‐care.

**Design:**

A cross‐sectional survey. The study included nurses working in units where HF patients were hospitalized.

**Results:**

The average score for comfort of education was 5.43 (between “slightly comfortable” and “very comfortable”). The most comfortable topics were “Daily weight monitoring” (5.81 ± 1.25), “Signs/symptoms of worsening condition” (5.77 ± 1.19) and “Fluid restriction” (5.76 ± 1.23). The respondents felt least comfortable when teaching about “Medications” (5.06 ± 1.35) and “Low‐sodium diet” (5.31 ± 1.42). The mean score obtained for education frequency was 5.21 (*SD* 2.51). The nurses most frequently educated their patients on such topics as “Daily weight monitoring” (5.82), “Signs/symptoms of worsening condition” (5.9) and “Fluid restriction” (5.92).

**Conclusions:**

Polish nurses are not ready to perform comprehensive HF care tasks without preparation.

## 1 BACKGROUND

Heart failure (HF) is a public health problem that affects 26 million people worldwide. Nearly 6 million Americans suffer from HF, and this number is expected to increase by 46% in 2030 (Rasmusson, Flattery, & Baas, 2015).

In Poland, the number of people with HF exceeds 750,000. Recent data from the Polish National Health Fund show that the public healthcare sector treats 650,000 people a year (Nessler, Kozierkiewicz, & Gackowski, 2019). It is estimated that this number will increase by up to 25% in the coming years.

The prevalence of HF is gradually increasing across much of the world. This is caused by the ageing of society and advances in the treatment of and survival from cardiovascular diseases (CVD). HF is associated with poor prognosis and high morbidity, which usually leads to hospitalization. Even though the diagnostics and treatment of HF have become highly advanced, adult patients are still at a considerable risk of death from this disorder. One‐year survival in the mild‐to‐moderate HF is 80%–90%, with severe HF—the rate stands at 50%–90% (Rice, Say, & Betihavas, 2018).

HF patients are usually readmitted because their discharge planning is insufficient and they are poorly educated with respect to HR, lack social support, do not have a partner and live alone, are not provided with the continuation of care, do not comply with their medication regime and demonstrate poor adherence to the instructions received (Betihavas et al., 2015; Howie‐Esquivel & Spicer, 2012; Luttik, Jaarsma, Moser, Sanderman, & van Veldhuisen, 2005). Due to their poor education and mistaken idea about the condition, HF patients may inadequately or unconfidently use their self‐care skills. Numerous authors have shown that self‐care intervention produces significantly better treatment outcomes in HF patients than the lack of such behaviours (Barnason, Zimmerman, & Young, 2012; Seto et al., 2011; Shao & Yeh, 2010).

The improvement of self‐management skills, resulting from disease management programmes, has yielded beneficial effects in adult HF patients, especially with respect to their well‐being, functioning, symptoms, morbidity and prognosis. According to practice guidelines, patient education concerning a heart‐healthy lifestyle remains a crucial element of effective disease management in adults with HF (HFSA et al., 2010; McMurray et al., 2012; NHFA CSANZ et al., 2018). It is very important that patients who are actively engaged in self‐care understand their disease and treatment regime (Mahramus et al., 2013). Studies have shown that patient education efficiently and cost‐effectively prevents rehospitalizations and that patients' knowledge about self‐care (e.g. symptom monitoring, medication compliance, diet observance) yields positive outcomes (Pressler, 2011; Simmonds et al., 2015). Nurses play a crucial role in providing patients and their families with education and preparing the former for postdischarge management of their disease.

There are several evidence‐based educational topics and nursing‐care performance measures which should be taken into account when managing HF: managing and recognizing symptoms, monitoring weight, diet, level of activity, medication and follow‐up appointments. To adequately educate patients about self‐care, nurses should have a comprehensive knowledge of HF and self‐care behaviours such as managing and maintaining the control of the disease and symptoms (Albert et al., 2002).

According to Orem's theory, engagement and self‐care skills may significantly reduce the need for hospitalization in cardiac patients (Cottin et al., 2004). The degree of patients' preparation depends on their ability to participate in their care. In the supportive‐educational model, patients are able to engage in self‐care but require education about the various aspects of therapeutic self‐care behaviours (Orem, 2001). Nurses are in a perfect position not only to recognize the existing and potential health problems but also to offer supportive‐educational interventions if necessary.

Some of the available study results show that nurses are not adequately prepared for education and knowledge of self‐care principles is insufficient to teach patients (Albert et al., 2002; Simmonds et al., 2015). These deficits assessing the level of nurses' preparation to educate HF patients and usually include signs and symptoms of hypoperfusion hydration status and blood pressure assessment, symptom management and diet and medication restrictions. It is believed that too little time is devoted for patient education in hospitals (Albert et al., 2002). Patients do not know enough about heart failure and treatment recommendations.

The ordinance published by the Polish Minister of Health has defined the main goal and specific objectives of the comprehensive heart failure care (Polish, Kompleksowa Opieka nad Osobami z Niewydolnością Serca—KONS). The main goal is to “limit the occurrence of consequences of HF,” while the specific objectives are as follows: early discovery of HF and determination of its aetiology, inhibiting disease progression, optimizing the use of healthcare resources in HF care, improving lives and longevity of patients with HF and limiting the exacerbation of the disease (Balsam et al., 2018; Nessler, Kozierkiewicz, et al., 2018; Nessler, Straburzyńska‐Migaj, et al., 2018; Nessler, Windak, & Oleszczyk, 2015). The proposed model of care will offer multidisciplinary, continuous and coordinated care, aiming to engage the patient in his or her treatment. It aims to deliver a comprehensive scope of care by combining outpatient care, pharmacology, interventional treatment (invasive cardiology, electrotherapy, cardiac surgery) and rehabilitation.

These teams will provide the necessary services: medical care, nursing care, education, drug administration and monitoring of patient compliance with physicians' advice. Consultant cardiologists will offer ongoing support to general practitioners in diagnosing and treating patients with HF. Education of patients about self‐care carried out by nurses should translate to a lower number of acute decompensated HF episodes and, consequently, slower the progression of the disease.

In the light of the previously demonstrated idea of slow delivery coordinated care for patients with heart failure and knowledge deficits in Polish nurses, which might lead to difficulties in delivering adequate patients education, we decided to assess the frequency of patient education provided by nurses before discharge and the level of comfort they experience when fulfilling the tasks of educators, preparing patients for self‐care.

*The aim of the study was to* assess education frequency and nurses' comfort when educating patients hospitalized in different hospital units to prepare them for self‐care. The following questions were posed: (a) Do nurses perform tasks related to the delivery of pre‐discharge patient education among all patients and/or their families? (b) Do nurses feel comfortable in the role of educators before discharge planning? (c) What determinants affect the frequency of education and nurses' level of comfort when delivering patient education?

## 2 METHODS

### 2.1 Study design and sample

This was a cross‐sectional study performed in the public and university hospital setting with the use of a descriptive design and survey method. The study included nurses working in units where HF patients were hospitalized: conservative cardiology, invasive cardiology, cardiac surgery and emergency units and cardiac rehabilitation units (those which provide care to HF patients).

Inclusion criteria:

- part‐ and full‐time Registered Nurses with medical secondary school education, bachelor's degree or master's degree
- professional duties involving HF patient care
- consent to participate in the study

Exclusion criteria:

- employment as a nurse administrator, nurse manager or a float Registered Nurse.

All nurses included in the study received information about the purpose of the study, its voluntary nature and its risks and benefits. They were also informed that participation in the study may increase their knowledge of HF. Signed informed written consent was obtained from all participating individuals. The invitation to participate in the study had been sent to 503 nurses employed mainly in cardiac units; only 304 accepted.

The final number of 304 nurses were asked to fulfil the Survey of RNs about Heart Failure Practices Related to Delivery of Patient Education before Discharge. Additionally, information on the participants' characteristics (age, gender, education, postgraduate training) and workplace (type of hospital, type of unit) was obtained. The analysis of the surveys collected revealed that all the nurses included in the study fulfilled the questionnaire on the frequency and comfort of delivering pre‐discharge patient education.

The study was approved by the Bioethics Committee at the Medical University.

Tool:

The Survey of RNs about Heart Failure Practices Related to Delivery of Patient Education before Discharge, authored by Nancy Albert, consists of questions divided into three parts:

Part I pertains to the general characteristics of nurses and their workplace.
Part II contains general questions about educating patients (the use of teaching resources such as videos and handbooks, time spent educating patients, contact with patients' family members, feelings related to comfort and confidence while delivering education). Answers are provided on a 5‐point Likert scale (ranging from “never” to “always”).
Part III is composed of questions about the most common topics discussed during heart failure education (illness beliefs, medications, low‐sodium diet, physical activity, weight monitoring, fluid restriction, symptoms of fluid overload). In this part, respondents determine the following:
  1. the level of their comfort while discussing HF topics using a 7‐point Likert‐type scale, where 1 denotes “completely uncomfortable” and 7 “completely comfortable,”
  2. the frequency of education about a given theme using a numerical scale from 0 (“I never discuss this topic”)–10 (“I always discuss this topic”).

We obtained the author's permission to use the questionnaire.

### 2.2 Statistical analysis

Comparisons of quantitative variables in two groups were conducted with Mann–Whitney test. Comparisons of quantitative variables in more than two groups were conducted with Kruskal–Wallis test. Dunn's test was used as post hoc procedure. Correlations between quantitative variables were assessed with Spearman's correlation coefficient. Analyses were conducted at the 0.05 level of significance.

## 3 RESULTS

Most of the group studied were women (90.79%); the mean age of the respondents was 42.55 ± 10.03 years. A large proportion of the nurses had higher education (74%) but did not undergo postgraduate training (only 25% of the nurses had a specialty). More than half of the respondents were employed at a university hospital (52.96%), in a telemetry unit (34.21%) and in an intensive cardiac care unit (25.33%). The nurses usually worked 12‐hr shifts (day–night). Work experience in the study group was 19.41 ± 11.34 years (Table 1).

**TABLE 1:** Characteristics of the study group

Variable	Values
Age	
Mean ± *SD*	42.55 ± 10.03
Median	45
Work experience	
Mean ± *SD*	19.41 ± 11.34
Median	21
Type of hospital	
City hospital	55 (18.42%)
Provincial hospital	87 (28.62%)
University hospital	161 (52.96%)
Gender	
Female	276 (90.79%)
Male	28 (9.21%)
Workplace	
Medical unit/ICU	47 (15.46%)
Emergency care/short‐stay unit	36 (11.84%)
Cardiac care area/telemetry unit	104 (34.21%)
Cardiac care unit/intensive care	77 (25.33%)
Cardiac surgery unit	21 (6.91%)
Cardiac rehabilitation unit	19 (6.25%)
Shift	
Usually day shift weekday	88 (28.95%)
Usually off‐shift weekday	27 (8.88%)
Usually weekend	1 (0.33%)
Equal rotation between days and off‐shifts	180 (59.21%)
PRN or parent shift	8 (2.63%)
Education	
Secondary	88 (28.95%)
Higher	216 (71.05%)
Specialties	
Yes	77 (25.33%)
No	227 (74.67%)

### 3.1 **Nurses**'** comfort: Survey of RNs about Heart Failure Practices Related to Delivery of Patient Education before Discharge**

The level of comfort was assessed using a 7‐point Likert‐type scale, where 1 denoted “completely uncomfortable” and 7 “completely comfortable,” based on the mean score for all the questions. The mean score obtained by the respondents was 5.43 (*SD* = 1.13). This means that the nurses felt between “slightly comfortable” and “very comfortable” in the role of educators (Table 2). As for the level of comfort in educating patients about individual topics, the respondents felt most comfortable with regard to “Daily weight monitoring” (5.81 ± 1.25), “Signs/symptoms of worsening condition” (5.77 ± 1.19) and “Fluid restriction” (5.76 ± 1.23) (Table 3). The mean score of nearly 6 suggests a high level of comfort when teaching about these topics. The respondents felt least comfortable when teaching about “Medications” (5.06 ± 1.35) and “Low‐sodium diet” (5.31 ± 1.42). In this case, the mean score was slightly over 5, which “merely” denotes “slightly comfortable.”

**TABLE 2:** Nurses' comfort in and frequency of delivering education—overall score—based on the Survey of RNs about Heart Failure Practices Related to Delivery of Patient Education before Discharge

	Mean	*SD*	Median	Min.	Max.	Q1	Q3
Level of comfort	5.43	1.13	5.53	1	7	4.86	6.32
Education frequency	5.21	2.51	4.91	0	10	3.59	7.11

* Eight nurses did not complete this questionnaire.

**TABLE 3:** Nurses' comfort when delivering education about individual topics

Topic	N*	Mean	*SD*	Median	Min.	Max.
Medications	290	5.06	1.35	5.1	1	7
Low‐sodium diet	295	5.31	1.42	5.29	1	7
Physical activity	296	5.46	1.27	5.67	1	7.67
Fluid restriction	295	5.76	1.23	6	1	7
Signs/symptoms of worsening condition	295	5.77	1.19	6	1	7
Daily weight monitoring	294	5.81	1.25	6	1	7
Signs/symptoms of fluid overload	295	5.45	1.27	5.5	1	8
HF illness beliefs	295	5.56	1.22	5.83	1	7

* Means that the number of nurses answering individual questions was different.

The themes (questions) below have been listed in descending order based on the mean score obtained: from the highest mean (the highest level of comfort) to the lowest mean (the lowest level of comfort). The respondents felt the highest level of comfort (the score of c. 6 on the Likert‐type scale) when educating about the importance of monitoring oneself and the need for accurate daily weight monitoring and measuring and monitoring fluid intake. The nurses experienced the lowest level of comfort (score below 5) when teaching about sexual activity and taking particular groups of medications. What is worth noting is that the respondents did not obtain the highest possible level of comfort in any of the questions (Table 4).

**TABLE 4:** Nurses' comfort in and frequency of delivering education about individual themes

Variable	Level of comfort			Education frequency		
	*N* *	Mean	*SD*	*N* *	Mean	*SD*
Why it is important to monitor self	295	5.86	1.19	284	6.29	2.61
Why weight self every day	294	5.86	1.26	286	6.24	2.62
Procedure for weighting self	293	5.84	1.34	280	6.22	2.68
What counts as fluids	295	5.82	1.24	281	6.17	2.65
When to notify someone of worsening condition	295	5.79	1.27	281	6.15	2.69
When to report weight to doctor/nurse	292	5.76	1.34	285	6.14	2.6
What to report when speaking to doctor/nurse	292	5.76	1.34	287	6.14	2.57
Steps to measure and monitor fluid intake	295	5.75	1.29	281	6.11	2.69
Why exercise	294	5.73	1.38	283	6.09	2.71
“What to look for” signs/symptoms of worsening condition	295	5.73	1.25	287	6.07	2.61
Why restrict fluids	295	5.71	1.34	286	6.01	2.61
How often to monitor signs/symptoms	295	5.71	1.26	284	6.01	2.66
Heart failure can be controlled by lifestyle actions	295	5.62	1.29	283	5.89	2.64
Regular office visits are important even when feeling fine	294	5.62	1.31	280	5.79	2.66
Types of exercises/activities recommended	295	5.61	1.37	285	5.78	2.57
What does “heart failure” mean	295	5.59	1.3	287	5.76	2.73
Why taking aspirin	290	5.58	1.5	280	5.74	2.56
Heart failure is chronic/debilitating	295	5.55	1.28	285	5.74	2.54
When to notify someone of increasing fluid levels	294	5.54	1.33	283	5.74	2.6
What to do if you become fatigued while exercising	295	5.52	1.37	278	5.71	2.61
What causes heart failure	295	5.51	1.35	279	5.67	2.63
Types of exercises/activities to avoid	295	5.5	1.38	268	5.66	2.67
Why maintain a 2,000 mg/day sodium diet	294	5.49	1.56	282	5.63	2.63
Why taking warfarin, if in atrial fibrillation	290	5.46	1.57	287	5.62	2.72
“What to look for” fluid overload	295	5.46	1.33	266	5.61	2.66
How to tell when exercising too much	295	5.45	1.36	278	5.57	2.71
Heart failure may shorten life/cause premature death	295	5.44	1.37	283	5.57	2.65
Why taking a loop diuretic (such as furosemide)	290	5.43	1.5	282	5.46	2.66
How to read a food label	294	5.4	1.53	283	5.45	2.71
How often to monitor for fluid overload	295	5.4	1.37	269	5.39	2.46
Why it is important to monitor for fluid overload	293	5.39	1.42	272	5.36	2.69
How to decrease sodium when at relatives	292	5.36	1.55	270	5.34	2.7
How to decrease sodium intake when snacking	293	5.34	1.56	276	5.28	2.74
How to identify sodium in packaged foods	294	5.25	1.57	276	5.24	2.78
Expected effects of taking a beta‐blocker	290	5.24	1.52	261	5.22	2.59
How to decrease sodium intake at a restaurant	293	5.23	1.57	269	5.19	2.76
What salt substitutes are OK to use	294	5.12	1.66	270	5.15	2.75
Why taking a beta‐blocker	290	5.02	1.51	260	5	2.59
Adverse effects from taking a beta‐blocker	288	5.02	1.57	265	4.99	2.48
Sexual activity	294	5.02	1.6	263	4.78	2.49
Why taking digoxin	290	4.91	1.59	254	4.77	2.58
Adverse effects of taking an ACE‐I or ARB	289	4.65	1.66	255	4.73	2.5
Why taking an aldosterone inhibitor	289	4.64	1.62	268	4.72	2.82
Why taking an angiotensin receptor blocker (ARB) or angiotensin‐converting enzyme inhibitor (ACE‐I)	290	4.61	1.57	262	4.71	2.55

* Some of the respondents skipped different parts of the questionnaire.

### 3.2 Education frequency: Survey of RNs about Heart Failure Practices Related to Delivery of Patient Education before Discharge

Education frequency was assessed using a numerical scale ranging from 0 (“I never discuss this topic”)–10 (“I always discuss this topic”) (every point denotes further 10% in education frequency). The mean score obtained by the respondents was 5.21 (*SD* 2.51), which means that about 52% of patients under their care were educated about all the topics listed in the questionnaire (Table 2). The nurses most frequently educated their patients on such topics as “Daily weight monitoring” (5.82), “Signs/symptoms of worsening condition” (5.9) and “Fluid restriction” (5.92). The mean scores of nearly 6 indicate that education about these topics was provided to approximately 60% of patients under the respondents' care (Table 5).

**TABLE 5:** Frequency of education about individual topics

Topic	*N* *	Mean	*SD*	Median	Min.	Max.
Medications	297	4.49	2.69	4.2	0	10
Low‐sodium diet	297	4.87	2.85	4.71	0	10
Physical activity	297	5.16	2.73	5	0	10
Fluid restriction	297	5.92	2.78	6	0	10
Signs/symptoms of worsening condition	297	5.9	2.74	6	0	10
Daily weight monitoring	297	5.82	2.85	6	0	10
Signs/symptoms of fluid overload	297	5.39	2.8	5	0	10
HF illness beliefs	297	5.54	2.72	5.17	0	10

* Some of the respondents skipped different parts of the questionnaire.

The least frequent education topics were “Medications” (4.49) and “Low‐sodium diet” (4.87). In this case, the mean scores were below 5, which means that fewer than 50% of the respondents' patients received education on these topics (Table 6).

**TABLE 6:** Frequency of education about individual topics and themes listed in the questionnaire

Variable	Education frequency		
	N*	Mean	*SD*
What counts as fluids	284	6.29	2.61
When to notify someone of worsening condition	286	6.24	2.62
What to report when speaking to doctor/nurse	280	6.22	2.68
Why restrict fluids	281	6.17	2.65
When to report weight to doctor/nurse	281	6.15	2.69
Steps to measure and monitor fluid intake	285	6.14	2.6
Why it is important to monitor self	287	6.14	2.57
Procedure for weighting self	281	6.11	2.69
Why weight self every day	283	6.09	2.71
What to look for fluid overload	287	6.07	2.61
How often to monitor signs/symptoms	286	6.01	2.61
Regular office visits are important even when feeling fine	284	6.01	2.66
Heart failure can be controlled by lifestyle actions	283	5.89	2.64
When to notify someone of increasing fluid levels	280	5.79	2.66
What does “heart failure” mean	285	5.78	2.57
Why exercise	287	5.76	2.73
What to look for medication	280	5.74	2.56
What causes heart failure	285	5.74	2.54
Heart failure is chronic/debilitating	283	5.74	2.6
Why it is important to monitor for fluid overload	278	5.71	2.61
How often to monitor for fluid overload	279	5.67	2.63
Why taking aspirin	268	5.66	2.67
Heart failure may shorten life/cause premature death	282	5.63	2.63
Types of exercises/activities recommended	287	5.62	2.72
Why taking warfarin, if in atrial fibrillation	266	5.61	2.66
Why maintain a 2,000 mg/day sodium diet	278	5.57	2.71
Types of exercises/activities to avoid	283	5.57	2.65
How to tell when exercising too much	282	5.46	2.66
What to do if you become fatigued while exercising	283	5.45	2.71
Why taking a loop diuretic (like furosemide)	269	5.39	2.46
How to decrease sodium intake when snacking	272	5.36	2.69
How to decrease sodium when at relatives	270	5.34	2.7
How to read a food label	276	5.28	2.74
How to identify sodium in packaged foods	276	5.24	2.78
Expected effects of taking a beta‐blocker	261	5.22	2.59
What salt substitutes are OK to use	269	5.19	2.76
How to decrease sodium intake at a restaurant	270	5.15	2.75
Adverse effects from taking a beta‐blocker	260	5	2.59
Why taking a beta‐blocker	265	4.99	2.48
Why taking digoxin	263	4.78	2.49
Adverse effects of taking an ACE‐I or ARB	254	4.77	2.58
Why taking an aldosterone inhibitor	255	4.73	2.5
Sexual activity	268	4.72	2.82
Why taking an angiotensin receptor blocker (ARB) or angiotensin‐converting enzyme inhibitor (ACE‐I)	262	4.71	2.55

Just as with the comfort level assessment, the nurses gave the lowest scores to the topic concerning the aim and adverse effects of taking HF medications. In this case, the respondents' score was below 5, which means that the topic was taught less than half of the time. The topics most frequently taught by the respondents included fluid restriction, weight monitoring and signs/symptoms of worsening condition, and the need for regular office visits. The results of the analysis demonstrated that over 60% of patients received education on these topics. Table 6 presents the themes (questions) listed in descending order based on the mean score obtained: from the highest mean (the highest education frequency) to the lowest mean (the lowest education frequency).

### 3.3 The relationship between selected determinants and comfort and frequency of education

The analysis of correlations between selected variables and the comfort in and frequency of delivering HF patient education demonstrated a significant positive relationship between the age of the respondents and nurse comfort (*r* = .166) and education frequency (*r* = .123). In other words, the older the nurses, the greater the comfort in and frequency of delivering education. A similar positive correlation was observed between work experience and nurse comfort; that is, the longer the experience, the higher the comfort in delivering education (*r* = .134). The analysis of the relationship between gender and the comfort in and frequency of delivering education revealed a statistically significant difference between male and female nurses. *p*‐values lower than .05 indicate that the comfort in and frequency of delivering education were significantly higher in the group of female nurses (Table 7).

**TABLE 7:** Impact of selected variables on the comfort in and frequency of delivering education

Parameter	Comfort				Frequency			
	Correlation coefficient	*p* *	Direction	Strength	Correlation coefficient	*p* *	Direction	Strength
Age	0.166	*p* = .004 NP	Positive	Very weak	0.123	*p* = .034 NP	Positive	Very weak
Work experience	0.134	*p* = .021 NP	Positive	Very weak	0.107	*p* = .065 NP	‐‐‐	‐‐‐

	Mean ± *SD*	Median	*p* *	Mean ± *SD*	Median	*p*
Gender						
Female	5.5 ± 1.06	5.61	.016 NP	5.34 ± 2.48	4.98	.026 NP
Male	4.8 ± 1.59	5		4.14 ± 2.48	4.19	
Type of hospital						
City hospital—A	4.92 ± 1.62	5.18	<.001 NP B > A,C	4.6 ± 2.84	4.58	.001 NP B > A,C
Provincial hospital—B	5.85 ± 0.93	5.93		5.93 ± 2.62	5.84	
University hospital—C	4.76 ± 0.94	5		4.23 ± 1.82	4	
Workplace						
Medical unit/ICU	5.39 ± 0.98	5.43	0.024 NP	4.9 ± 2.4	4.43	0.008 NP
Emergency care/short‐stay unit	4.78 ± 1.63	5.18		4.32 ± 3.02	4.49	
Cardiac care area/telemetry unit	5.44 ± 0.91	5.41		5.23 ± 2.04	4.64	
Cardiac care unit/intensive care	5.71 ± 1.15	6.09		5.66 ± 2.73	5.52	
Cardiac surgery unit	5.54 ± 0.91	5.61		4.85 ± 2.35	5	
Cardiac rehabilitation unit	5.75 ± 0.82	6		7 ± 2	7.8	
Education						
Secondary	5.43 ± 1.05	5.32	0.708	4.97 ± 2.57	4.56	0.202
Higher	5.44 ± 1.17	5.63	NP	5.31 ± 2.49	5	NP
Specialty	5.84 ± 0.78	5.98	0.001	5.8 ± 2.42	5.6	0.015
None	5.3 ± 1.2	5.39	NP	5.01 ± 2.51	4.64	NP
Specialty in cardiac care	6.29 ± 0.62	6.32	<0.001	7.08 ± 2.28	6.89	0.001
Other specialty	5.36 ± 1.14	5.45	NP	5.06 ± 2.47	4.77	NP

* P = normal (parametric) distribution in groups, ANOVA + post hoc analysis results (Fisher's LSD test); NP = non‐parametric distribution in groups, Kruskal–Wallis test + post hoc analysis results (Dunn's test).

The post hoc analysis of correlations between the type of hospital and nurses' comfort in and frequency of delivering education showed that employees at provincial hospitals experienced significantly higher comfort than those employed at city and university hospitals; also, the former group delivered education with significantly higher frequency (Table 7).

As for correlations with the respondents' workplace, the highest comfort in delivering education was observed in employees of the cardiac rehabilitation unit (5.75 ± 0.82) and cardiac intensive care unit (5.71 ± 1.15). Similar results were obtained with regard to education frequency, which was the highest in the cardiac rehabilitation unit (70% of patients), followed by the cardiac intensive care unit (56% of patients) and the telemetry unit (52% of patients). The lowest comfort was experienced by nurses in the emergency care/short‐stay unit (4.78 ± 1.63), who also demonstrated the lowest frequency of delivering education (43% of patients).

The analysis of the relationship between the respondents' university education and the comfort in and frequency of education delivery showed that this was not a variable that had a significant impact on the parameters studied. The opposite applies to postgraduate training as nurses with a specialty demonstrated a significantly higher level of comfort and education frequency than those without a specialty (5.84 ± 0.78 versus 5.3 ± 1.2; *p* = .001 for comfort; 5.8 ± 2.42 versus 5.01 ± 2.51; *p* = .015 for frequency). As for the type of specialty, the analysis showed that the respondents with a specialty in cardiac care presented a significantly higher level of comfort and delivered pre‐discharge education to HF patients more frequently than those with any other specialty (6.29 ± 0.62 versus 5.36 ± 1.14 for comfort; 7.08 ± 12.28 versus 5.06 ± 2.47 for frequency).

## 4 DISCUSSION

Heart failure is a complicated clinical entity leading to patient's premature death. The appropriate management of heart failure includes pharmacotherapy, non‐pharmacologic treatment and early recognition of decompensation. Every deviation of optimal treatment methods contribute to the steady or rapid worsening of the patient's status, which in turn activates the neurohumoral systems wasting the failing heart's resources and shortening life expectancy. The knowledge about optimal heart failure management is crucial for the patient to live longer, and any kind of measures or anyone who may contribute to better disease and symptom understanding will influence the prolongation of patients' life.

According to the guidelines, patient education is of immense importance. Also, patients should receive relevant training materials as part of their pre‐discharge education (Paul, 2008). The American Nurses Association (ANA) Scope and Standards of Cardiovascular Nursing emphasize that the primary responsibility for delivering patient education rests with nurses (ANA, 2015). Having analysed 29 trials of multidisciplinary management programmes, McAlister et al. concluded that a programme is successful if it incorporates nurses with enough knowledge on HF (McAlister, Stewart, Ferrua, & McMurray, 2004). Nurses working in cardiac units play a crucial role in assessing the response of patients to the treatment plan (with modifications if necessary) and preparing patients and their families for postdischarge self‐care. One of the meta‐analyses demonstrated that patients who received pre‐discharge educational interventions were more likely to weigh themselves daily, observe and implement sodium restrictions and avoid smoking compared with patients receiving standard care. Pre‐discharge targeted HF education delivered to patients results in a reduced number of days hospitalized, decreases mortality rate and enhances self‐care behaviours (Koelling, Johnson, Cody, & Aaronson, 2005; Kramers, de Mulder, Barth, & Wagener, 1993). Studies by other authors showed that hospital readmission was reduced in HF patients who had received educational intervention from a specialist nurse. Also, there is evidence of improved outcomes in severely disabled HF patients who, together with their families, had been educated by a specialist nurse. This is why it is so important for cardiac nurses to understand the difficulties patients face with respect to self‐care and adherence and pursue teaching strategies that will allow patients to cope with their problems (Stamp, Machado, & Allen, 2014).

According to the authors of the curriculum for heart failure, it is the geographical location and professional regulations in a given state that determine the role and duties of a nurse (Riley et al., 2016). On the other hand, other works indicate that nurses have the need to deliver high‐quality pre‐discharge education, but the lack of time and knowledge deficits make it difficult for them to do so (Hart, Spiva, & Kimble, 2011; Mahramus et al., 2013, 2014; Vreeland, Rea, & Montgomery, 2011; Willette, Surrells, Davis, & Bush, 2007).

We examined in our study mostly nurses with higher level of education—relatively with long professional experiences and from university hospitals.

The most important result from our study was that the overall comfort score indicated that the nurses felt rather comfortable educating HF patients. The respondents felt most comfortable when teaching about such topics as daily weight monitoring, fluid restriction and signs/symptoms of worsening condition. On the other hand, the nurses' comfort was lowest with respect to delivering education on medications and low‐sodium diet. The lowest scores were observed, in particular, for the aim and adverse effects of taking medications and sexual activity issues. The same applies to the frequency of education; that is, the topics mentioned as most comfortable to the nurses were also most frequently taught by them. According to Delaney et al., nurses demonstrate merely basic knowledge about HF symptoms (Cottin et al., 2004). However, in our own study, the correlation analysis demonstrated that nurses' comfort in and frequency of delivering education depended on their level of knowledge and university and postgraduate education. The topics that were most frequently taught and where nurses felt most comfortable are also the most common areas of cardiac nursing research. In their nursing research analysis, Stamp et al. conclude that nurse‐led interventions and patient education are a common area of research, but what should be examined is method analysis, timing, duration and the impact of these activities on the outcome. Besides, there are only few nursing studies (Stamp et al., 2014) investigating questions related to treatment, its adverse effects, decompensated HF management and biomarker evaluation pointing to poor clinical outcomes and low QoL (Stamp et al., 2014). The results obtained by Mahramus et al. point to significant deficits in nurses' knowledge about signs/symptoms of HF, its aggravation, the assessment of blood pressure and fluids, management of symptoms and restrictions related to diet and medications (Simmonds et al., 2015).

It is worth noting that the patients included in Mahramus' study displayed a very low level of detailed knowledge on the effects of HF medications and the importance of fluid restriction. It is necessary point out that our study demonstrated that nurses rarely taught patients about these topics and felt uncomfortable while doing it. What follows is that the problem of knowledge deficits will continue to exacerbate and translate into an unsatisfactory level of self‐care our educated patients.

Despite the common knowledge that education significantly improves treatment outcomes, educational interventions are not consistently incorporated into practice. The second important result of our research showed that the overall score for the frequency of patient education was about 52% and the least frequently discussed topics were low‐sodium diet and medications. This is in line with the results in other studies (Basuray et al., 2015). Stamp and colleagues demonstrated that nearly 45% of patients never or very rarely received 60 min of instructions, which stands in contrast to the guidelines which recommend this amount of time for pre‐discharge self‐care education (Stamp et al., 2014). Our study did not assess the time spent on education delivery but focused on the frequency of education.

Our results showed that nurses who worked in the cardiac intensive care unit experienced higher level of comfort and presented higher frequency of delivering education than nurses from the medical floor. The highest level of comfort and education frequency was observed in nurses working in the cardiac rehabilitation unit. This is a surprising result, but our data are not consistent with those presented in the literature and it may result from the specifics of sanatorium care and continuing hospital treatment in rehabilitation wards. According to Linge, nurses who did not work in acute care felt more strongly that they devoted over 30 min, on average, to pre‐discharge education compared with those working in acute care who spent less than 30 min delivering pre‐discharge education. The results we obtained can be explained by the current standards of employment in Poland, or rather lack of such standards. The number of patients per every nurse is distinctly higher in medical units than in other units where the study was conducted, except for the emergency care unit. Hospitalization time is also short. Moreover, cardiac units rarely employ nurses whose main responsibility is patient education. In contrast, in cardiac rehabilitation units, patient education is a fixed element of a usually 4‐week‐long stay, with clearly defined educational tasks delegated to the entire therapeutic team. Finally, our study showed that nurses working in the emergency unit felt the lowest level of comfort in education delivery among the respondents and provided education least frequently, that is only to 43% of patients. Numerous authors claim that pre‐discharge education delivered by a specialist emergency nurse is crucial to high‐quality care. This is because there are situations where HF patients do not know whether to seek additional assistance when experiencing new/ongoing symptoms. It is difficult to identify the cause of such a low score in our study. Maybe it stemmed from poor work organization and insufficient number of personnel, or from knowledge deficits and inadequate training of the nurses in the role of medical educators. In emergency units, patients and their families may not show interest in education due to symptom exacerbation and problem‐solving orientation demonstrated by the personnel. Some researchers believe that it is better to provide education to patients in a stable condition, who have adapted to living with HF (Paul, 2008).

Another factor affecting the comfort in and frequency of education delivery was the type of hospital. Nurses in provincial hospitals felt the highest level of comfort and provided education with the highest frequency, whereas employees at university hospitals obtained the lowest scores in this respect. This comes as a surprise because one might think that nurses working in university facilities perform educational tasks regularly and that teaching should not present any serious difficulty to them. One possible cause of such a low level of nurse comfort and education frequency is that university hospitals often employ graduates in nursing and, according to the results of our correlation analysis, age and work experience had the highest impact on comfort and frequency, whereas university education did not affect these parameters. Unfortunately, we did not obtain information on the respondents' preparation for the role of educators. It is likely that a large proportion of nurses do not go through the required period of occupational adaptation and do not receive internal training in a given workplace. It should be emphasized that our study did not assess nurses' knowledge and level of preparation for the role of educators teaching HF patients about self‐care, but it is very likely that the low level of nurse comfort and education frequency is not related to knowledge deficits as regards the topics discussed. So far, no one in Poland has conducted research assessing knowledge and preparing nurses to educate patients.

Nurses educating HF patients should receive relevant training so that the entire staff convey consistent educational information (Rasmusson et al., 2015). Educational tasks based on the HF guidelines should be treated as a priority. It is important to treat patients and their families as partners rather than students in the educational process (Paul, 2008). Educational interventions should take the form of two‐way sessions, with patients taking an active part in the identification of their needs.

There are researchers who believe that lack of time is the most commonly reported reason for insufficient education (Baas et al., 2014; Peter et al., 2015). Other health system‐related barriers include lack of support from decision‐makers, difficulties with handling electronic documentation and lack of educational materials. It is possible that all these data are the basis for assessing the effectiveness of education, but they were not the subject of this work.

Our study demonstrated that nurses' postgraduate training significantly affected the comfort in and frequency of delivering education. Nurses with a specialty degree felt a higher level of comfort and delivered education more frequently than those without a specialty. Additionally, the values of these parameters were higher in nurses with a specialty in cardiac care than in nurses with other specialties. Previous research has shown that Polish nurses demonstrate a much lower level of knowledge about self‐care principles than their counterparts in Europe and the world (Jankowska‐Polańska, Brzykowska, Uchmanowicz, Lisiak, & Rosinczuk, 2017). This might partially be explained by the general nature of nursing education they receive and the fact that nurses do not pursue cardiac nursing specialty degree or courses (Jankowska‐Polańska et al., 2017). Formal HF certification equips nurses with advanced knowledge to be conveyed to patients, fosters their credibility and accountability and gives them a sense of accomplishment as an integral component of HF care (Altman, 2011; Buonocore, Trupp, & Wingate, 2013).

Our study also demonstrated that the comfort in and frequency of pre‐discharge HF education delivery were affected by gender, age and work experience of nurses. Female and older nurses experienced higher comfort and provided patient education with higher frequency than male and younger nurses. Also, nurses with longer work experience felt higher comfort in educating patients. It is difficult to explain these correlations. There are no studies in the existing literature which would analyse the relationship between these variables and the parameters we investigated. In the previous Polish studies, age was not a factor that affected nurses' level of knowledge. However, the study by Kalogirou demonstrated that age and long work experience correlated with the lower level of knowledge, especially among nurses working in outpatient health care (Fowler, 2012; Jankowska‐Polańska et al., 2017). The authors explained this low level of knowledge by low participation of older nurses in postgraduate training. In our study, as much as 74% of the respondents had higher education and 25% participated in specialty training. This clearly shows that the number of nurses holding a master's degree has grown considerably in the recent years in Poland. Furthermore, in the last couple of years, the governmental programme of nurse qualification improvement allowed practising nurses to participate in specialty courses free of charge, which could certainly have influenced the results obtained in our study. In their study, Albert et al. (2002) demonstrated that American nurses with a training certificate, master's degree and longer work experience in HF care may present better decision‐making, especially with respect to the management of medications for chronic conditions and acute care.

### 4.1 Clinical and practical perspectives

These authors emphasize the need for developing systems supporting nurses, including the following: programmes with an appropriate curriculum which would improve nurses' HF self‐care knowledge, motivation for nurses to pursue HF specialty, sufficient number of staff and time that would allow for pre‐discharge education, streamlined educational plan documentation and outcome evaluation and incorporation of comprehensive patient education into overall HF care.

### 4.2 Study limitations

Our research has a few limitations. It is essential that there is no control over how the questionnaire is filled in. Nurses could consult with each other and use other sources of knowledge to conduct the survey. In the organization of coordinated care for patients with heart failure, primary care is given an important role in the care process and only clinical nurses have participated in our study, which may not reflect the reality of the situation. Another limitation of the study is the lack of verification of work experience and courses by survey fillers and uneven group selection in terms of academic education (75% of nurses had higher education). Another limitation of the survey is the lack of information about the reasons for refusing to participate in the survey and information about which nurses did not fill in the questionnaires (personal and professional profile).

## 5 CONCLUSION

1. Polish nurses are not ready to perform comprehensive heart failure care tasks under CONS, without proper, careful preparation.
2. Male and female nurses' comfort in educating HF patients is higher than the frequency of education delivered.
3. The lowest level of nurse comfort and education frequency was observed with respect to the following topics: HF treatment, aim and adverse effects of taking particular groups of medications, sexual activity of patients and principles of low‐sodium diet.
4. Factors affecting the comfort in and frequency of education delivery are nurses' age, work experience, gender, type of hospital and postgraduate training.
5. Completion of a cardiac specialty significantly affects nurses' comfort in and frequency of delivering education about self‐care to HF patients.

## CONFLICT OF INTEREST

The authors declare that they have no conflict of interest.

## AUTHOR CONTRIBUTIONS

DK and BJP were responsible for the conception and design, acquisition of data, analysis and interpretation of data, drafting the initial manuscript and revising it critically for important intellectual content. DK wrote the manuscript. All authors read and approved the final manuscript.

## ETHICAL APPROVAL

The study protocol was approved by the Independent Bioethics Committee of the Wroclaw Medical University (Approval No. KB 205/2019). All participants gave informed consent after thorough explanation of the procedures involved. The study was carried out in accordance with the tenets of the Declaration of Helsinki.

## CONSENT FOR PUBLICATION

All co‐authors have agreed to the submission and publication of this manuscript.

## Data Availability

All data and materials used in this research are freely available. References have been provided.
